# Reemergence of Rift Valley Fever, Mauritania, 2010

**DOI:** 10.3201/eid2002.130996

**Published:** 2014-02

**Authors:** Ousmane Faye, Hampathé Ba, Yamar Ba, Caio C.M. Freire, Oumar Faye, Oumar Ndiaye, Isselmou O. Elgady, Paolo M.A. Zanotto, Mawlouth Diallo, Amadou A. Sall

**Affiliations:** Institut Pasteur, Dakar, Senegal (O. Faye, Y. Ba, O. Faye, M. Diallo, A.A. Sall);; Institut National de Recherche en Santé Publique, Nouakchott, Mauritania (H. Ba, I. O. Elgady);; University of São Paulo, São Paulo, Brazil (C.C.M. Freire, P.M.A. Zanotto)

**Keywords:** Rift Valley fever, Rift Valley fever viruses, viruses, outbreak, virus lineage, reemergence, field investigations, human, animals, mosquitoes, Mauritania

## Abstract

A Rift Valley fever (RVF) outbreak in humans and animals occurred in Mauritania in 2010. Thirty cases of RVF in humans and 3 deaths were identified. RVFV isolates were recovered from humans, camels, sheep, goats, and *Culex antennatus* mosquitoes. Phylogenetic analysis of isolates indicated a virus origin from western Africa.

Rift Valley fever (RVF) is an acute febrile viral disease that affects domestic ruminants and humans. The disease in animals is characterized by abortions among pregnant females and high mortality rates for offspring (*1*). The causative pathogen, RVF virus (RVFV), is a member of the family *Bunyaviridae*, genus *Phlebovirus*, and its genome consists of 3 RNA segments: large (L), medium (M), and small (S) (*2*). In human infections, symptoms are generally mild but may evolve to severe symptoms, such as hemorrhaging, meningoencephalitis, and retinopathy, with fatal outcomes (*3*). RVF is endemic to sub-Saharan Africa, Egypt, Saudi Arabia, and Yemen (*4*). In western Africa, a major RVF outbreak occurred in Mauritania and Senegal in 1987 and resulted in 220 human deaths (*5*). This outbreak was followed by epizootics/epidemics in 1998 and 2003 in different provinces in Mauritania (Hodh El Gharbi, Assaba, Brakna, Trarza, and Gorgol Provinces) (*6*,*7*).

In October 2010, the health services of Adrar Province in Mauritania were informed of the deaths of 2 girls with hemorrhagic syndromes and 2 persons with acute fever, arthralgia, and headaches in Amogjar Province. Blood samples collected from a sick patient were sent to Institut Pasteur in Dakar, Senegal where RVFV IgM and RVFV genome were detected. During October 28–November 11, 2010, suspected RVF cases were identified in several cities Adar Province (Atar, Aoujeft, Chinguetti), Inchiri Province (Akjoujt), and Hodh el Gharbi Province (Tintane, Kobeni) (Figure 1), and high mortality rates among camels, sheep, and goats were reported in Adrar and Inchiri Provinces (*8*). Clinical samples from humans and animals with suspected RVF were positive for this virus.

**Figure 1:**
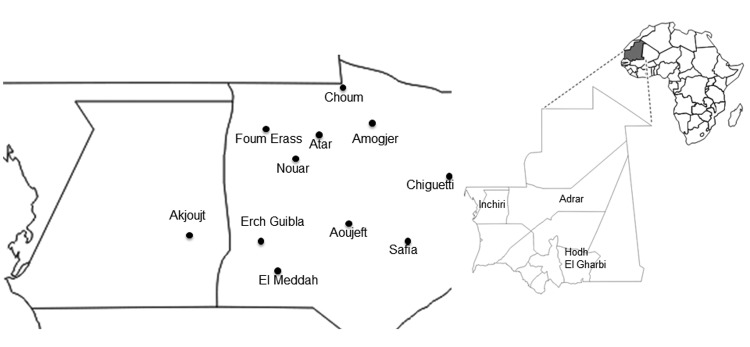
Location of study sites tested for patients with confirmed Rift Valley fever, Mauritania, 2010.

These cases led to identification of 2 additional confirmed cases of acute RVF (positive for IgM against RVFV or RVFV RNA) in Adrar and Inchiei Provinces. Multidisciplinary investigations involving epidemiologists, virologists, entomologists, and veterinary teams in areas where the cases occurred were conducted in December 2010 as part of other investigations (*8*), describe the extent of the outbreak, and investigate possible RVF reemergence factors in Mauritania. We report results of laboratory and field investigations among humans, animals, and mosquitoes during the 2010 outbreak.

## The Study

During October–December 2010, surveillance and investigation teams collected 80 clinical samples from 36 humans with suspected RVF and 44 of their contacts in Atar, Aoujeft, Akjoujt, Chingetti, Kobeni, and Tintane Moughatas (Figure 1). A human with a suspected case of RVF was defined as any patient with fever associated with or without hemorrhage, jaundice, or neurologic symptoms during October–December. A human contact was defined as a healthy person living within the immediate environment (i.e., same household or neighborhood) of a human or animal with a confirmed case of RVF. Serum samples from suspected case-patients were tested by using real time reverse transcription PCR, ELISA (for detection of IgM), and virus isolation.

Samples were obtained from animals living near suspected or confirmed case-patients, and a questionnaire about the species, sex, age and reproductive status of female animals was completed by their owners. Sampling of mosquitoes was conducted in December 2010 by using animal-baited traps placed in and around houses in which suspected and/or confirmed case-patients were identified. RVFV isolation and identification was performed on mosquitoes and human serum samples as described (*9*). Coding regions for RVFV nonstructural protein and glycoprotein 2 were amplified by using reverse transcription PCR and described sets of primers (*10*,*11*) and then purified after electrophoresis on agarose gels, and sequenced for phylogenetic analyses.

IgM against RVFV or viral RNA genome was detected in 30 (37.5%) of 80 patients, and 6 RVFV isolates were recovered (Table 1). The dates of symptom onset for confirmed cases of RVF in humans indicated that the outbreak probably started at the end of October, peaked in November, and decreased the second week of December. Among 30 patients (8 contacts and 22 suspected case-patients) with acute RVFV infections, the male: female ratio was 5:1 (25:5), and the median age was 35 years (range 17–70 years); 3 deaths were reported. Except for 1 man (a teacher), all infected men were herdsmen and all infected women were housewives.

**Table 1:** Seroepidemiologic and virologic results for humans, Rift Valley Fever outbreak, Mauritania, 2010*

**City**	**No. tested**	**No. positive for IgM against RVFV**	**No. positive for RVFV by RT-PCR**	**No. with isolated virus**
**Atar**	54	12	10	**3**
**Aoujeft**	12	1	7	**3**
**Akjoujt**	10	0	4	**0**
**Chinguetti**	2	0	1	**0**
**Kobeni**	1	0	0	**0**
**Tintane**	1	0	0	**0**
**Total**	80	0	22	**6**

*RVFV, Rift Valley fever virus; RT-PCR, reverse transcription PCR.

For the animal investigation, serum samples were obtained and processed from 83 small ruminants (70 goats and 13 sheep) with a median age 4 years (range 1–10 years) from all localities tested (Table 2, Appendix). IgM against RVFV was detected in 23 (27.7%) of 83 animals. In addition, samples from 5 sick camels were collected in neighborhoods of persons with suspected and confirmed cases of RVF, and RVFV RNA was detected in 3 of these samples. Information for populations living in Adrar indicated that the first probable case of the disease among animals was in a sick camel in Aoujeft during the last week of October (*8*).

**Table 2:** Serologic and virologic results for animals, Rift Valley Fever outbreak, Mauritania, 2010*

**Animal**	**No. females**	**No. abortions**	**No. positive for IgM against RVFV**	**No. positive for RVFV by RT-PCR**
**Sheep**	13	8	5	**0**
**Goats**	70	19	18	**0**
**Camels**	5	0	0	**3**
**Total**	88	27	23	**3**

*RVFV, Rift Valley fever virus; RT-PCR, reverse transcription PCR.

A total of 2,741 mosquitoes belonging to 5 genera and 11 species were collected for entomologic investigation. *Culex antennatus* was the most abundant mosquito species (83%), followed by *Anopheles gambiae (*9%) and *An. rufipes* (2%) mosquitoes (Table 3). Three RVFV strains were isolated from *Cx. antennatus* mosquitoes collected in Safia (Adrar Province).

**Table 3:** Virologic results for mosquitoes tested during a Rift Valley fever outbreak, Mauritania, 2010*

**Mosquitoes species **	**No. tested**	**No. mosquito pools**	**No. mosquitoes positive for RVFV by RT-PCR**	**No. with isolated virus**
***Aedes vexans***	13	3	0	0
***Anopheles gambiae***	259	10	0	0
***Anopheles pharoensis***	51	7	0	0
***Anopheles rufipes***	47	7	0	0
***Anopheles pretoriensis***	3	1	0	0
***Anopheles gambiae***	36	2	0	0
***Anopheles pharoensis***	2,276	46	4	4
***Anopheles rufipes***	16	4	0	0
***Anopheles pretoriensis***	30	5	0	0
**Total**	2,736	85	4	3

*RVFV, Rift Valley fever virus; RT-PCR, reverse transcription PCR.

**Figure 2:**
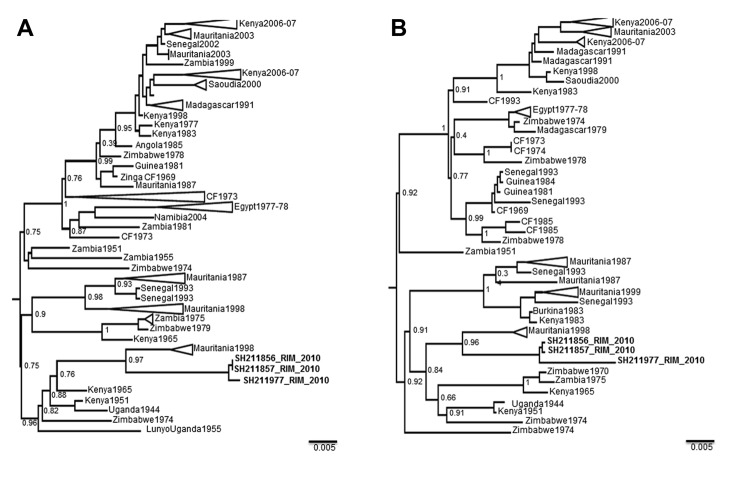
Maximum-likelihood trees for the RNA A) small and B) medium segments of Rift Valley fever virus. Tees show relationships among strains isolated from different localities and countries. Samples from Mauritania 2010 are indicated in **boldface** and designated SH211856_RIM_2010, SH211857_RIM_2010, and SH211977_RIM_2010. GenBank accession nos. are KF717588, KF717589, and KF717590 for the medium segment and KF717591, KF717592, and KF717593 for the small segment. Values along the branches indicate bootstrap values. Scale bars indicate nucleotide substitutions per site.

Phylogenetic analysis based on RVFV partial small and medium RNA sequences showed a low level of variation between isolates from humans, animals, and mosquitoes. Isolates belonged to the West Africa lineage and were closely related to strains from Diawara (1998) and Hodh El Garbi (1998) in Mauritania and from Kenya (1965), Zimbabwe (1974), and Zambia (1975) (Figure 2).

## Conclusions

After a 4-fold increase in rainfall in Mauritania from 2009 through 2010 (59 mm vs. 196 mm; (Aoujeft Weather Station). RVF clinical cases were confirmed in Adrar and Inchiri Provinces. Although RVF was reported in some provinces of Mauritania in 1987, 1998, and 2003 (*5*–*7*), cases in humans from Adrar and Inchiri Provinces have not been previously reported. However, in 2010 a total of 30 laboratory-confirmed human case-patients were recorded in these 2 provinces; 3 of the case-patients died. Both areas had a drought in recent years or their average rainfall was not >60 mm before 2010. The study showed animal abortion rates of 26.13% (23/88) and 30.68% (27/88) in 2 groups of small ruminants living near patients with recent RVFV infections. Three camels were infected with RVFV, and the cases were probably linked to human infections. The first human death from RVF was reported on November 11 in the village of Tawaz, in which a high mortality rates were observed in camel populations (*11*).

Serologic evidence of RVF in camels from northern Sudan and southern Egypt in 1977 (*12*) led to camels being suspected as key contributors in RVFV transmission (*13*). The involvement of camels in human infections could explain the high male:female ratio (25:5) for infections in humans because men are more involved in animal husbandry. In 2006, a similar sex difference for infection was observed in Kenya, where men had a prevalence rate of IgG against RVFV that was 3 times higher than that in women (*14*).

Entomologic investigations in Mauritania showed that only *Cx. antennatus* mosquitoes were infected with RVFV. Similar finding were reported for previous RVF outbreaks in Mauritania. Furthermore, *Cx. antennatus* mosquitoes were found to be infected with RVFV in Nigeria in 1967 and in Kenya during 1981–1984, and vector competence was demonstrated in laboratory studies (*15*). Phylogenetic analysis suggests reemergence of the local RVFV focus and emphasizes the need to strengthen RVF surveillance and other control measure in Mauritania after heavy rainfall.
